# “Give me the knowledge, and I can do what I want with it, it’s my right and my choice”: Triangulated perspectives on the disclosure of young onset dementia

**DOI:** 10.1177/14713012231191958

**Published:** 2023-07-28

**Authors:** Siobhán Fox, Tony Foley, Suzanne Cahill, Caroline Kilty

**Affiliations:** Centre for Gerontology and Rehabilitation, School of Medicine, 8795 University College Cork, Cork, Ireland; Department of General Practice, 8795 University College Cork, Cork, Ireland; School of Social Work and Social Policy, 8809 Trinity College Dublin, Dublin, Ireland; Centre for Economic and Social Research on Dementia, NUI Galway, Galway, Ireland; Institute of Gerontology, Jonkoping University, Jonkoping, Sweden; Catherine McAuley School of Nursing and Midwifery, 8795 University College Cork, Cork, Ireland

**Keywords:** diagnosis, disclosure, young onset dementia, carer, healthcare professional, frontotemporal dementia, qualitative research

## Abstract

**Introduction:**

Receiving a diagnosis of young onset dementia is particularly distressing; the person under 65 years is often in employment, with financial commitments, young children, and an active social life. Some of the stress experienced by younger people experiencing cognitive changes can be reduced by an early and accurate diagnosis, but this is contingent on the timing of disclosure and a process which is sensitive and appropriate to the person. The study aim was to explore experiences of giving and receiving a diagnosis of young onset dementia, by triangulating the perspectives of the key parties involved.

**Methods:**

A qualitative design was employed, using semi-structured interviews. Participants (*N* = 47) included people with young onset dementia (*n* = 10), family members (*n* = 12), and health and social care professionals (*n* = 25). Thematic analysis and triangulation enabled identification of overall themes across different participant groups.

**Results:**

All participant groups agreed on key aspects of good disclosure practice, with two overarching themes: The optimal conditions for disclosure, and how best to disclose a diagnosis. Positive experiences of disclosure were prefaced on having the appropriate space and time; having a support person present; clearly labelling the diagnosis; providing appropriate information at the right pace. Other findings include recommendations for longer appointment times, offering additional support for young families, and for carers of people with atypical presentations (e.g. frontotemporal dementia).

**Conclusion:**

Many people with young onset dementia had unsatisfactory disclosure experiences. Health and social care professionals should provide a ‘pre-disclosure’ appointment, elicit the amount of information the person may want at the point of disclosure of the diagnosis, balance truth and hope, provide contact details for follow-up, and overall be mindful of the individual in front of them. While young onset dementia may be a life-altering diagnosis, a disclosure meeting which is sensitively undertaken can increase the person’s agency, coping ability, and ultimately empower them to live well with their diagnosis.

## Introduction

Young onset dementia refers to the onset of dementia before the age of 65 (Koopmans and Rosness, 2014). Receiving a diagnosis of dementia can be distressing at any age, but the impact on younger people, who are often in employment, with young families, greater financial commitments, and active social lives, is particularly profound. Worldwide, there are 3.9 million people aged 30–64 years living with young onset dementia (Hendriks et al., 2021). In Ireland, young onset dementia has been reported to account for up to 10% of all cases of dementia (Pierce et al., 2014).

People with young onset dementia often have a challenging pathway to diagnosis. A range of diseases cause young onset dementia. While Alzheimer’s disease is the most common dementia overall, it accounts for only about one third of all cases in young onset dementia compared with about two thirds in older people (Kwon and Lee, 2017; O’Malley et al., 2019). The occurrence of certain sub-types including fronto-temporal dementia and alcohol-related dementia is much higher in younger people (Jefferies and Agrawal, 2009; Koopmans et al., 2013). Atypical presentations of dementia are also more common in younger people, for example the dominant presenting problem may not be memory related, but a change in mood, personality, or behaviour (Rossor et al., 2010). This can lead to clinical under-investigation, misdiagnosis, and significant delays in receiving diagnosis (Carter et al., 2018). Indeed, compared to late onset dementia, the average time from symptom onset to diagnosis may be up to two-three times as long at 4.4–4.7 years (Draper et al., 2016; Novek et al., 2016; O’Malley et al., 2019). Compared with their older counterparts, people with young onset dementia may have heightened awareness of their cognitive deficits (van Vliet et al., 2013), increasing stress and anxiety in the lead up to and during the diagnostic process. Family members living with a person with young onset dementia also experience significant stress during this time, relating to difficulty managing non-cognitive symptoms, the long quest for diagnosis, nondisclosure of problems to others, and denial of diagnosis (Ducharme et al., 2013).

The stress and anxiety faced by people experiencing cognitive changes can be somewhat mitigated by a timely, accurate, and sensitive diagnosis (Poyser and Tickle, 2019). Better awareness of dementia and increasing diagnostic rates are regarded generally as national and international health priorities (DOH, 2014; WHO, 2012) and represent key objectives of most countries’ national dementia strategies (Cahill, 2020; Pot and Petrea, 2013; WHO, 2017). However, more recently the importance of how the diagnosis is disclosed is increasingly recognised. Disclosure is the evolving process of sharing a diagnosis with a patient, including how and when it is shared (Frank et al., 2018). Although younger people receiving a diagnosis of dementia are likely to be more significantly impacted, they remain under-represented in conversations on this topic. Moreover, guidelines for clinicians disclosing a diagnosis of dementia are lacking, with a dearth of guidance for young onset dementia. A recent publication (O’Malley et al., 2021b) produced a series of statements to inform best practice in receiving a diagnosis of young onset dementia. However, just 3 of the 27 statements related to the disclosure process.

Three key parties are involved in diagnosis disclosure of young onset dementia: the health and social care professional(s) delivering the diagnosis, the individual receiving the diagnosis, and (usually) a family member or other support person. Previous research has typically considered each of these groups separately, and moreover the voice of the person with young onset dementia remains vastly under-represented (O’Malley et al., 2021a). To fully understand the process of diagnosis disclosure in young onset dementia, it is important to elicit the perceptions of each of these groups. In a systematic review and meta-ethnography of the literature on diagnosis disclosure of dementia in general and not young onset dementia, Poyser and Tickle (2019) identified five key themes exploring the experience of the disclosure of a dementia diagnosis from the perspectives of clinicians, patients, and carers, namely: the clinician’s approach; how to tell people the diagnosis is dementia; the importance of the clinician offering hope; level of understanding; and who should attend the disclosure meeting. However, only 3 of the 13 included studies involved all 3 stakeholders, with just 1 of these adopting a clearly articulated triangulation approach to analysis, and only 2 of the studies included a small proportion of people aged under 65 years. A more recent scoping review of the qualitative literature on the self-reported experience of receiving a diagnosis of young onset dementia specifically, uncovered just 8 studies (O’Malley et al., 2021a). Again, much attention was given to the initial pathway to diagnosis, although two themes around the disclosure process were identified, namely, the language used, and the reaction to the diagnosis (O’Malley et al., 2021b). While it is promising to see research emerging in this area, a greater understanding of this complex process is needed.

A qualitative methodology involving triangulation of the experiences of key stakeholders is required to deepen our understanding of what is a complex and personal healthcare issue. The aim of this study was to explore the experiences of giving and receiving a diagnosis of young onset dementia, by triangulating the perspectives of people with young onset dementia, health and social care professionals, and families. The research question was: What factors relate to a successful disclosure meeting for the diagnosis of young onset dementia?

## Methods

### Design

A qualitative design was used, employing semi-structured in-depth interviews. A topic guide was created, and interview schedules were devised for each participant cohort (see supplemental file 1). The schedules were developed in discussion with an advisory group of clinical and topic area experts, including people with young onset dementia, family carers, advocacy representatives, policy-makers, expert academics, and health and social care professionals of various disciplines, to ensure that they adequately addressed the research question and were appropriately structured for people with young onset dementia. The first interview in each sub-group was treated as a pilot, however no changes to the topic guide were made.

### Sampling and recruitment

A maximum variation purposive sampling technique (Etikan, et al., 2016) was used to recruit a small but diverse range of people with young onset dementia and family members. Study invitation packs were distributed via the Alzheimer Society of Ireland, regional Memory Clinics, and via national social media channels. Potential participants “opted-in” by contacting the research team. Further study information was provided, and basic demographic details were collected over the phone for the researchers to select a diverse sample in terms of young onset dementia sub-type, relationship of the family member, age, geographic spread etc. We sought those more recently diagnosed, who may have better ability to recall their diagnosis. The inclusion criteria were: a person with young onset dementia, or a family carer of a person with young onset dementia, diagnosed in the previous 5 years.

Using maximum variation purposive sampling, health and social care professionals were recruited from healthcare services across five regions in Ireland; these sites included Memory Clinics, community-based dementia services and primary care centres. The sites were chosen to represent a geographical spread, and included two cities, two towns also serving a large rural population, and one rural site. A gatekeeper identified at each site was responsible for advertising the study to their colleagues and staff. Inclusion criteria for health and social care professionals were: involved, directly or indirectly, at any stage of the diagnostic process for young onset dementia; >6 months experience in this field.

### Data collection

All interviews were conducted by two researchers experienced in dementia and qualitative research (CK and SF). All interviews were audio-recorded (with permission), transcribed verbatim, and identifying details such as names were replaced with codes.

People with young onset dementia were given the choice of completing an interview individually, or with a companion. Dyadic interviews were closely monitored to ensure no individual experience was overlooked. The inclusive method of dyad interviewing aligns with relationship-centred approach to care (Soklaridis et al., 2016). All interviews with people with young onset dementia and family members were conducted face-to face in a location convenient to them (e.g. their home, or a neutral location). Interviews lasted an average of 72 minutes (range = 32–120 minutes). Health and social care professionals were given the option of completing the interview face-to-face in their workplace, or over the phone. All were individual interviews, lasting 40 minutes on average (range = 10–72 minutes).

In total, 47 individuals participated in 40 interviews (Table 1), including 25 health and social care professionals (all individual interviews; by phone *n* = 23, in-person *n* = 2), 10 people with young onset dementia and 12 family members (across 8 individual interviews and 7 dyadic interviews, all in-person).

**Table 1.** Demographic details of participants.

Category	N	Category	N	Category	N
People living with young onset dementia	10	Family members	12	Health and social care professionals	25
Gender		Gender		Gender	
Female	6	Female	10	Female	20
Male	4	Male	2	Male	5
Dementia sub-type		Relationship to person living with young onset dementia		Profession	
Young onset dementia (sub-type unknown or unspecified)	6	Spouse	8	Geriatrician	4
Alzheimer’s disease	2	Child	4	General practitioner	3
Logopenic progressive aphasia	1			Clinical nurse specialist - dementia	3
Lewy body dementia	1			Occupational therapist	3
				Dementia advisor	2
				Neurologist	2
				Advanced nurse practitioner	2
				Dementia coordinator	2
				Nurse manager	1
				Staff nurse – Memory clinic	1
				Neuropsychologist	1
				Social worker	1

### Ethical Considerations

Appropriate guidelines for conducting psychosocial research with people with dementia were followed, including the Alzheimer Europe position paper (Gove et al., 2018) and key publications relating to ethical issues, participatory research, and active involvement of people with dementia in research (Bartlett and Martin, 2002; Dewing, 2007, 2008; Hellstrom, et al., 2007; McKeown, et al., 2010). All participants were fully informed about the study’s aims and objectives, and the voluntary nature of participation and scope to withdraw, prior to obtaining written consent. Prior to the commencement of the interview the researchers re-visited the topic of consent, the objectives of the research, and offered participants the opportunity to ask questions. People with young onset dementia and family members were provided with contact information for relevant supports. Approval was granted by the social research ethics committee at University College Cork (ref:2019-077).

### Data analysis

Both interviewers agreed on data saturation when no new themes were emergent in interviews, although an additional 2-3 interviews were completed per stakeholder as confirmation. NVivo software was used to support data management and analysis, which took an iterative approach. First, the datasets from the three participant cohorts were analysed separately, and key themes were identified within each. This followed the Thematic Analysis framework of Braun and Clarke (2006); an example of the process of analytic steps is in Table 2. To conduct data analysis, the transcripts were read and re-read, notes on initial observations were made. When becoming more familiar with the data, labels, or codes, were assigned to areas of importance. Tentative themes were constructed from these codes via a process of contextual decision making, and these themes were again reviewed by cross-checking against the dataset before being determined. Themes were identified at a semantic level, i.e. the themes were identified within the explicit or surface meanings of the data. Next, the data were triangulated to address completeness, convergence, and dissonance of key themes from the separate datasets (Farmer et al., 2006). Two authors were involved in coding, co-coding a proportion of the transcripts to ensure consistency and credibility. A recursive rather than linear approach was followed to allow for scope to move back and forth between steps as the analytic process warranted. Findings were discussed with the advisory group.

**Table 2.** Example of thematic analysis process.

Context	Main theme	Subtheme	Code	Excerpt
Interview question: “What was the experience of getting a diagnosis like, for you?”	How best to disclose a diagnosis to avoid harm	Clarity of the diagnosis	Lack of clarity	“Yeah well it kind of went over my head a bit at the start because they just said it wasn’t Alzheimer’s but it was a form of dementia so I didn’t realise it was the full thing you know”

## Findings

### Themes

**Figure 1.**
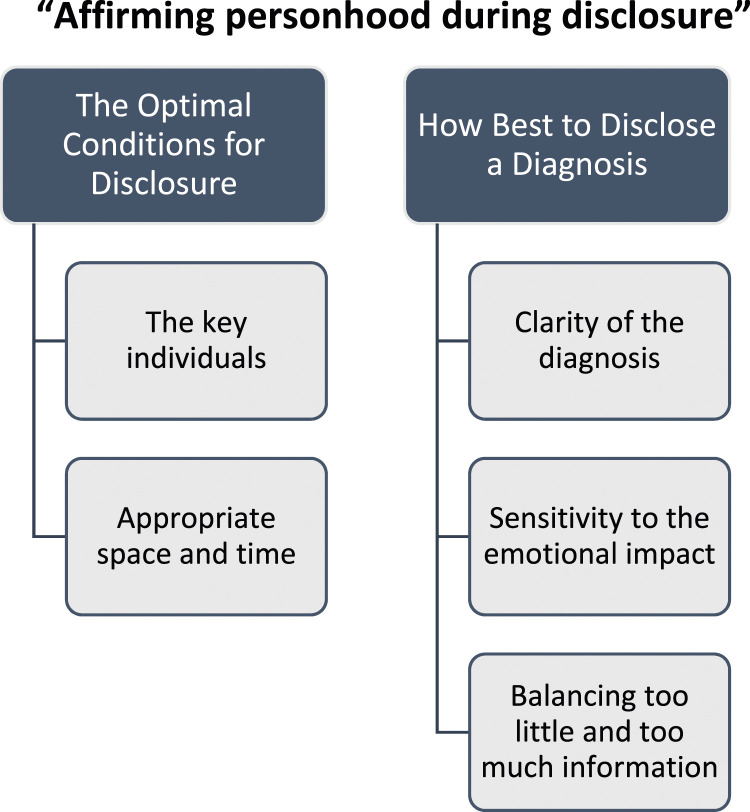
Diagram of themes and sub-themes.

There was consensus among people with young onset dementia, families, health and social care professionals, as to the importance and complexity of the disclosure process in young onset dementia. Two higher order themes and five sub-themes were identified from the triangulated dataset (Figure 1). An overarching theme, “affirming personhood during disclosure” captures the essence of all the main and sub-themes.

#### Theme 1: The Optimal Setting for Disclosure

##### The key individuals

All participant groups agreed that a person should be invited to have a supportive relative present for disclosure. Several people with young onset dementia and family members who received the diagnosis alone reported upsetting experiences, as in the following example of a person with young onset dementia and his wife, a couple in their 40s:

[Person with young onset dementia 7]: (the consultant) said to me then that…it was a form of dementia, I was by myself…I suppose when I was told that day in the hospital maybe more of an explanation there and then because it did go over my head a bit, if I was given more information as to what he was saying.

[Family member 9]: I was (abroad) at the time…I think if (he) had someone with him and if I had someone with me. You shouldn’t be alone for something like that…they told him when he was by himself.

Offering a different perspective, a family member described her experience of a disclosure meeting with her husband (not a participant) receiving a diagnosis of frontotemporal dementia while he did not seem to have insight into his own memory and behavioural difficulties and was experiencing significant psychiatric symptoms. She argued the full weight of the diagnosis fell on her and the rest of the family, and that in this instance, it would have been preferable for her to be afforded the opportunity to follow-up with the relevant health and social care professional alone.

I think if we had had a consultation before the diagnosis when (my husband) wasn’t there, we would have been able to ask questions. we didn’t get to do that. also, if there was someone there to speak to afterwards, to be able to go to and ask questions…[Family member 7]

In agreement with the views of people with young onset dementia and family members, most health and social care professional participants reported that they routinely invite the person to attend with a family member for the disclosure meeting. All had a policy of disclosing directly to the person with young onset dementia, although acknowledged that in some cases such as a very progressed dementia or in frontotemporal dementia, as in the above example, it may be necessary to talk mostly or sometimes separately to the family as well.

Health and social care professionals further elaborated on which professionals should be present, with many declaring that a small multi-disciplinary team of 2–3 is most suitable to deliver the diagnosis. In their experience, having too many health and social care professionals facing the family all at once is intimidating, thus in some practices not all of these health and social care professionals were present for the disclosure meeting, but were available in a room nearby should the person and family want more time after finishing with the doctor. While such multi-disciplinary team set-ups were typical at larger regional memory clinics, diagnosis by a general practitioner or by a consultant at a general clinic or in an acute hospital differed, potentially accounting for some of the poorer experiences reported by people with young onset dementia.

Unfortunately, we should have more personnel for the diagnostic process but it is very old school…for the diagnostic process it’s just a doctor. [Neurologist 1]

##### Appropriate space and time

Participants, including people with young onset dementia, family members, and health and social care professionals discussed the need for privacy, and the importance of having a quiet room when disclosing a diagnosis of young onset dementia. People with young onset dementia and family members who were not afforded this space reported poor experiences.

I was trying not to cry in front of the other people in the corridor. [Person with young onset dementia 5]

It was also important to the person with young onset dementia and family members that they were afforded enough time in the disclosure meeting:

This is life impacting stuff and it’s very robotic you know, next, next. It’s very rushed you know. And this is devastating stuff you know. It needs to be handled a bit better for all concerned. [Family member 4]

Ample space and time needed for a disclosure meeting was also acknowledged by health and social care professionals. Many felt that disclosing a diagnosis to a younger person requires more time than for an older person; in their experience the diagnosis may be more of a shock and those receiving it may have more questions and need more information. Those who had the facility to do so would schedule longer appointments for this age cohort where possible. Adequate staffing levels were important to ensure enough time can be allocated. One participant discussed a lack of time as a barrier to effectively diagnosing young onset dementia in their geriatric clinic versus their specialist clinic:

I can’t bring a 50-year-old to my clinic with 25 other 80-year-olds, they have a different set of problems and it takes more time. So you need somebody who can dedicate an afternoon to see five or six people in their 50s as opposed to my clinics where it’s 25 people in their 80s in an afternoon. [Geriatrician 1]

Some positive accounts of disclosure meetings were provided by a small number of people with young onset dementia and families. In one instance, where a diagnosis was made to a man and his wife with a very young family, a disclosure meeting was led by the neurology team within the hospital. A private setting was used, and key additional multidisciplinary colleagues attended to provide information and support.

They were well prepared for me, to be fair to them. They had everything prepared and they wanted me to understand what was happening and they wanted it all to sink in. They had a liaison officer there as well and I was put into the family room in the overflow ward…[Family member 9]

#### Theme 2: How best to disclose a diagnosis

##### Clarity of the Diagnosis

There was a difference of opinion between people with young onset dementia and family members, compared with health and social care professionals, on how clearly a diagnosis of young onset dementia was communicated. From the accounts of people with young onset dementia and family members, the different terms used to describe young onset dementia, the variants of young onset dementia, and a perceived reluctance on the part of the clinician in using the accurate term, caused a lack of clarity, and ultimately more distress. One person with young onset dementia shared her experience of attending the memory clinic and starting cholinesterase inhibitor medication, without feeling fully informed of her diagnosis.

(The memory clinic) asked would I be willing to take medication so I said of course because all that was on my mind was (my children). So I got home…I looked up the medication on my phone and the medication was for Alzheimer’s. So, none of that was conducive. It wasn’t a right way to do it. Now, no one used the word Alzheimer’s when I was there. [Person with young onset dementia 6]

For others, the sub-type of young onset dementia was named, but it was not fully understood by them that they had dementia.

Yeah well it kind of went over my head a bit at the start because they just said it wasn’t Alzheimer’s but it was a form of dementia so I didn’t realise it was the full thing you know [Person with young onset dementia 7]

Compounding the lack of clarity, some people with young onset dementia and family members reported that, even when the term dementia was used, they were given a “possible” or “probable” rather than a “definite” diagnosis. Another participant described the clinician’s reluctance to confirm the dementia, other than for required forms; this seemingly contradictory approach created ambiguity:

[Person with young onset dementia 3]: Yeah, so that was with (my daughter), in the memory clinic with (the consultant) took us and we were in the waiting room…and we went in and she said “It is dementia”.

[Family Member 5 (their daughter)]: No, she said “I have no diagnosis, but it is presenting as a dementia syndrome”. She didn’t give a diagnosis. But she said she could put it in writing for work, because she needed it. But she said that’s what it was presenting as.

Opposing opinions were common among health and social care professionals, most of whom in this sample felt that they were “getting better” at clearly using the word ‘dementia’. Some health and social care professionals detailed the steps taken to help a patient understand their diagnosis, with many also providing written details about the diagnosis.

We [i.e. the team members present] explain in plain English what we think is going on, and we name it. We say, “We think there is an Alzheimer’s process going on,” or, “We think that the vessels in the brain are affected and this is a vascular dementia.” So we name it, and they take it home with them in writing. [Social Worker 1]

However, another health and social care professional’s account of delivering a diagnosis reveals more of the reluctance to name young onset dementia, instead referring to the difficulties with diagnostic testing. This may help to explain the perceived lack of clarity expressed in the accounts of people with young onset dementia and family members:

We don’t just say ‘you have Alzheimer’s’, we say ‘look, we looked at your test, we looked at your scans, we looked at your history, we looked at the collateral history we got from your family, and you know we’ve out ruled this, we’ve out ruled that, we’ve done a lumbar puncture and this is what is showed’, and we explain to them that…no test is 100% accurate but the combination of all the tests is pointing towards this…and this is in our clinical opinion is what we think is wrong…because…there’s no 100% test in life for the majority of dementias. [Clinical Nurse Specialist 2]

##### Sensitivity to the emotional impact

Mixed accounts were expressed between and within stakeholder groups on the emotions experienced around disclosure of a diagnosis of young onset dementia. For some people with young onset dementia and family members, it was felt that clear communication and confirmation of diagnosis was a positive step, explaining the many changes they had been experiencing. As wait times can be long and stressful, sometimes finally getting to the diagnosis was a relief. Health and social care professionals identified similar positive responses from their patients. However, even for those who perhaps were expecting the diagnosis, it can still come as a shock:

Even though I partly guessed what it was it’s still a shock and it was even more of a shock for my husband like my husband couldn’t sleep for months. [Person with young onset dementia 5]

For others, the diagnosis was completely unexpected, and they experienced deep grief. Feelings of hope and hopelessness were recurrent in the people with young onset dementia and family member accounts of their disclosure experiences.

[Person with young onset dementia 2]: They had a consultant come in and he said “you have Alzheimer’s”. I had went for a brain scan before that and they had said there’s nothing wrong or there’s nothing growing or anything and the consultant said that he disagreed. They said it was fatal-

[Family member 2 (spouse)]: -Terminal

[Person with young onset dementia 2]: -Sorry terminal, that there was no cure. And it was put as bluntly as that. And at that stage you’re kind of going in to be cured and you’re coming out being told to put your affairs in order.

People with young onset dementia and families who were more prepared for the diagnosis, reported better experiences. A person with young onset dementia recounted how, prior to disclosure, the clinicians had a conversation with them on how much the person would like to know, and how much detail they would like provided.

There was a consultant and (another) doctor. He said ‘How do you want to play this, do you want the truth, the whole truth or nothing but the truth…or something else?’…We said the truth. [[Person with young onset dementia 2]

Many health and social care professionals tried to get a sense first of what the person felt was wrong, and how much insight they had, so that they could disclose the diagnosis at an acceptable pace, over several sessions where appropriate. Some health and social care professionals in memory clinics offered a ‘pre-disclosure appointment’ which was utilised to prepare patients for the possibility of a dementia diagnosis, with the intention of easing the shock at the diagnosis.

The patient comes in initially for the pre-assessment with our [Advanced Nurse Practitioner] and she would do the memory test…So it’s not as if somebody just walked in the door and been told, “You have a dementia.” That bit of groundwork is done for everybody regardless. They’re not just walking out of here thinking, “I’m fine.” [Nurse Manager 1]

Health and social care professionals expressed empathy and compassion, and many were acutely aware of the immense grief experienced by the person receiving a diagnosis, especially for a younger person. There was an emotional impact on the health and social care professionals themselves, who struggled with breaking this devastating news, particularly to patients they had known for many years.

There’s fierce loss and there’s all sorts of feelings come up, and people are devastated, so even though you’re doing your best for them, it’s a horrible thing to be telling them and people are devastated…we do our best, but it’s still what you’re telling them is devastating. [Clinical Nurse Specialist 3]

Some of the health and social care professionals talked about balancing hope while simultaneously giving people a realistic perception of dementia.

What we are trying to instil is a perspective of hope, just saying, “Look, this is a chronic disease.” We do say out loud, “There is no cure for it. It will get worse. But there is a medication which may help with the symptoms” [Social Worker 1]

Health and social care professionals experienced in diagnosis felt that communication style must be tailored to the patient. In their experiences, some people might ask straight out “*how much time they have*” and want a lot of information, others may initially be in shock and may not be able to take in much more than the word “*dementia*” and will need time to absorb this information before hearing more. Health and social care professionals felt that a good disclosure meeting will be paced to meet the needs of each individual, reflecting the people with young onset dementia’s accounts.

##### Balancing too little and too much information

When diagnosis was confirmed, several people and their family members stated that they received inadequate information, and were given little indication about where to turn next. One teenage son expressed upset at the lack of additional information or support offered with his parent’s diagnosis.

it’s just a horrible situation…it’s hard to think of a better setup but I guess if I was helped a lot more, instead of just left go…not just left…It was like almost getting told you have a cough. They said “you have dementia….see you later” basically you know they didn’t tell us about what we could do in the future [Family member 3]

The importance of provision of written information and detailed explanation was mentioned often, particularly by those who were not expecting the diagnosis. Furthermore, a gap in the information available was identified by several participants who received atypical young onset dementia diagnoses. For some, they reported getting no additional information about the type of young onset dementia at diagnosis, and resorted to seeking information themselves on the internet, and researching available supports themselves once they returned home.

We never got a leaflet, information sheet or any information, nothing leaving that hospital. Even if it’s something that you put in the drawer and look at a month later…We didn’t get any [telephone] numbers or anything…you spend so much time afterward ringing around here and there [Person with young onset dementia 7 and Family member 9]

A minority of participants shared examples of being provided with too much information during the disclosure meeting. These participants were advised to begin planning for the future, such as filing an Enduring Power of Attorney and organising a driving assessment. They would have preferred getting more detailed information about their diagnosis of young onset dementia and have time to assimilate this, and not what they might have considered peripheral matters at the time.

A divergence of opinion was apparent with the health and social care professionals, who typically reported that detailed verbal and written information is usually given out at the disclosure meeting, including information on the type of dementia, medication if being prescribed, and modifiable risk factors (i.e. diet, exercise, alcohol). Some health and social care professionals said they bring up legal issues such as future planning and wills, although the disclosure meeting was generally not felt to be the time to go as far as to discuss advance healthcare directives. Some gave information on other legal/lifestyle issues such as employment and financial help. Most discussed driving and the need for a driving assessment now or in the future, and recognised that this was a particular concern for their patients. Giving information on the medical tests that have been completed was reported to help people to accept the diagnosis. Health and social care professionals at memory clinics appeared to give more detailed information than other settings.

We have written information that is given to the patient…it goes through what we based our reasoning on, so is the summary of the history and the testing so there from the families and protesting in quite basic language we might mention the scans that we’ve done as well in the written information and we would say how certain we are of the diagnosis. Then we’d outline all these discussions around medication around future planning and then the follow-up as well that would be discussed. [Geriatrician 1]

Policies differed across the different settings. Some health and social care professionals had a standard information pack that is given to everyone diagnosed with young onset dementia, others had information packs that would be tailored to the individual. Others simply left the information leaflets on the desk and asked the person to take any they felt might be useful. Each clinic had their own reasons for doing it their way. For example, the clinic where leaflets were left out on the table had a dedicated health and social care professional providing phone follow-up after diagnosis so there was a further opportunity to impart important information.

While most health and social care professionals felt that providing written information about the diagnosis was important at the disclosure meeting, where there was a good local post-diagnostic support system, it was preferential to give further information at a slower pace over a few weeks by a clinic nurse who would remain linked in with the person and family. The importance of receiving timely and comprehensive information was articulated well by the following person with young onset dementia:

I really believe knowledge is power. So give me the information, give me the knowledge and I can do what I want with it, it’s my right and my choice. I really get angry when people struggle for information because it’s not a real resource demand. [Person with young onset dementia 6]

## Discussion

Most people with suspected dementia wish to know their diagnosis (van den Dungen et al., 2014). While in the past the disclosure to the person of a diagnosis of dementia was rare in Ireland (Cahill et al., 2006), today most health and social care professionals are committed to open disclosure. However, the process of disclosing a diagnosis of young onset dementia to the person and their family members is particularly complex. This study adds to the literature by triangulating the perspectives of the three key stakeholder groups involved in the process of disclosing a diagnosis of young onset dementia, and addresses our research question: What factors relate to a successful disclosure meeting for the diagnosis of young onset dementia?

The two main themes identified were “the optimal conditions for disclosure” and “how best to disclose a diagnosis”. Some of the experiences and suggestions for improvements shared herein overlap with those reported for late onset dementia (Poyser and Tickle, 2019), for example the importance of the clinician’s style of delivery and of offering hope, having the opportunity to ask questions, the importance of providing the right amount of information, and the benefit of having a carer present. There are also particular considerations of diagnosing young onset dementia; for example, the experience of diagnosis differed depending on the sub-type of young onset dementia. People with behavioural variant frontotemporal dementia are more likely than those with other dementia sub-types to have a complicated path to diagnosis, including more prior misdiagnoses of depression, bipolar affective disorder, or schizophrenia (Woolley et al., 2011), and to have limited insight into their condition (Griffin et al., 2016). Spousal and child carers of people with frontotemporal dementia experience significant burden (Kaizik et al., 2017). These factors should be taken into consideration by teams making a diagnosis of young onset dementia.

As with late onset dementia, while a good disclosure meeting should be directed at the person experiencing cognitive changes, family members should be considered and supported during the disclosure process. Spouses of people being diagnosed with young onset dementia experience particular stresses such as nondisclosure to others and denial of diagnosis, grief for loss of spouse and midlife projects (Ducharme et al., 2013). People with young onset dementia may have younger children who may experience embarrassment about their parent’s diagnosis and fear for the future (Millenaar et al., 2014). Extra support for these families through diagnosis disclosure is warranted. Notably, the few positive disclosure experiences reported herein related to diagnoses given to younger people with young onset dementia, with younger families. It is possible that even within young onset dementia ageism exists whereby a more considered disclosure meeting is afforded to someone in their late 40s or early 50s than those diagnosed with young onset dementia at an older age-future research would be needed to investigate this supposition.

Using a modified Delphi approach with 18 people with young onset dementia and 18 supporters, O’Malley et al. (2021b) developed consensus statements to inform best practice in diagnosing young onset dementia. The first of three statements relating to disclosure is that health and social care professionals should explain medical terms simply and clearly. In the current findings misleading and sometimes contradictory language was often used to convey information about the young onset dementia diagnosis, as reported by people with young onset dementia and families. Insights from health and social care professionals in this study elucidated that it was in effort to avoid distress, that they used mixed messages or euphemisms. Alternatively, as young onset dementia is difficult to accurately diagnose, and the many sub-types and different presentations compared to late onset dementia add to the complexity (O’Malley et al., 2019; Rossor et al., 2010), some health and social care professionals framed a diagnosis as “possible” or “likely” dementia during disclosure, which is confusing for people with young onset dementia and family members. This insight into disclosure practices from multiple stakeholder perspectives, helps to explain other’s reports of diagnosis disclosure of young onset dementia as traumatic (O’Malley et al., 2021a).

Further best practice in disclosing young onset dementia (O’Malley et al., 2021b) is that health and social care professionals should remember that receiving a diagnosis of young onset dementia is “a lot to take in”. A diagnosis of young onset dementia is much less expected than a diagnosis of dementia for older people (Greenwood and Smith, 2016), and the experience of shock may be universal, regardless of disclosure practices (Rabanal et al., 2018). This was borne out in the current results; receiving a diagnosis of young onset dementia was clearly very emotive, even for those who may have been expecting it. We noted that health and social care professionals who prepared people with young onset dementia and families for the diagnosis at a “pre-disclosure” meeting reported better experiences. Notably, the health and social care professionals were generally empathetic and committed to good disclosure practices. This corresponds with the literature on late onset dementia where health and social care professionals also face difficulties with disclosure and acknowledge personal emotional challenges in delivering a diagnosis (Milby et al., 2017).

O’Malley et al.’s (2021b) third and final consensus statement on disclosure of young onset dementia, reaching only 40% agreement, is that people should be provided with a letter including details of the diagnosis. Conflicting findings on the level of information wanted at diagnosis have been reported, with some people with young onset dementia feeling overwhelmed with information at diagnosis (Rabanal et al., 2018). The current findings suggest that information needs are very individual, but that the overall most pressing information needs at the time of disclosure are: information about the diagnosis, treatment, some key first steps, and a nominated point of contact. Those in employment will need particular information around their employment rights and financial supports, and may need to be signposted to government or financial advisory services (Kilty et al., 2023). As discussed above, the triangulation method unveiled some differences between stakeholder groups. Notably, in contrast to the accounts of people with young onset dementia and families, most health and social care professionals believed that they were competent and skilled at disclosing a diagnosis of young onset dementia. Importantly, not all of our participants with young onset dementia were diagnosed by teams represented by the health and social care professionals included in this study. Thus, this discrepancy may have arisen because the current sample of health and social care professionals may have had better practice than most, and disclosure practices are generally even poorer than reported here. There could also be discrepancy between how health and social care professionals perceive the disclosure meeting, and how well the person with young onset dementia and their families felt it went; health and social care professionals rarely receive feedback on this process.

It is also helpful to consider the overarching theme “affirming personhood during disclosure”. Crucial to an effective disclosure process was that the team making the diagnosis considered dementia as an experience that happens to a whole person. People with young onset dementia and their carer want to feel valued and respected as individuals in every interaction. Eliciting an understanding of a person’s and dyad’s unique relational style and personal information needs, and tailoring the disclosure of diagnosis, underlined the positive experiences herein.

### Strengths and limitations

Study strengths included that we recruited a varied sample, in terms of level of service use, young onset dementia sub-type, employment status, relationship of the family member to the person with young onset dementia, age. Our sample of health and social care professionals included geriatricians, neurologists, general practitioners, various nurse specialities and allied health professionals, a neuropsychologist, and a social worker. We included dyad and individual interviews, and ensured that the voice of the person with young onset dementia was well represented, addressing a gap in previous literature. We employed a triangulation method to increase validity and provide a clearer picture of the disclosure process in young onset dementia. We limited participants with young onset dementia and families to those diagnosed in the previous five years, however we still cannot be certain of the accuracy of participant’s recollections regarding disclosure. Most participants were female, reflecting the gender imbalance among both formal and informal carers. Phone interviews were a convenient method of recruiting a greater number of health and social care professionals, however they limit the ability to observe behaviour and body language. Finally, recruitment and social desirability biases mean that health and social care professionals may have reported better practices than in actuality.

### Implications for policy, practice and research

Although people with young onset dementia have much in common with people diagnosed with dementia in later life, receiving a diagnosis of dementia is arguably even harder for those with young onset dementia who have unique challenges. Through triangulating data from multiple key sources, this study highlighted specific areas which need addressing in an optimum disclosure meeting for young onset dementia. The current results attest that having appropriate space and time for disclosure, providing written information, having a close family member or trusted friend present, and the opportunity to ask questions are important to help the person absorb their diagnosis and scope out further the relevant information and advice needed. On the part of health and social care professionals, diagnosing young onset dementia is very complex, and sensitive disclosure practice requires great skill. Communication training should be a requirement for all health and social care professionals involved in disclosing a diagnosis of young onset dementia. An individualised approach on the part of the clinician is important, with the language and amount of information given tailored to the individual, with lots of opportunity for questions. Ideally both pre- and follow-up disclosure meetings should be offered. Further psychological supports surrounding disclosure should be resourced and made available, particularly to those diagnosed with rarer sub-types of young onset dementia.

### Conclusion

Regrettably, poor practices around the disclosure of a diagnosis of young onset dementia are common, and can have significant negative impact on the person receiving the diagnosis and their family. The current results provide insight into how disclosure practices may be improved, which may lead to benefits including increased sense of agency for the person with young onset dementia, lessened stress and anxiety for them and their family, and greater professional fulfilment for health and social care professionals.

## Supplemental Material

Supplemental Material - “Give me the knowledge, and I can do what I want with it, it’s my right and my choice”: Triangulated perspectives on the disclosure of young onset dementia Click here for additional data file. Supplemental Material for “Give me the knowledge, and I can do what I want with it, it’s my right and my choice”: Triangulated perspectives on the disclosure of young onset dementia by Siobhan Fox, Tony Foley, Suzanne Cahill, Caroline Kilty in Dementia.
