# Supplementary material for: “Give me the knowledge, and I can do what I want with it, it’s my right and my choice”: Triangulated perspectives on the disclosure of young onset dementia
Source: Dementia (London). 2023 Jul 28;22(8):1757–75. doi: 10.1177/14713012231191958 (PMC10644682; doi:10.1177/14713012231191958)
Supplement: Supplemental Material - “Give me the knowledge, and I can do what I want with it, it’s my right and my choice”: Triangulated perspectives on the disclosure of young onset dementia [file sj-pdf-1-dem-10.1177_14713012231191958.pdf]

Interview Topic Guides

Semi-structured interview schedule for <u>persons living with YOD.</u>

<u>Before diagnosis</u>

- Can you tell me about the time leading to diagnosis?
  - What were the symptoms or signs that made you first aware that something was wrong?
  - First steps, who did you tell, where did you go, etc?
  - How long did this process take? How did you feel about that…?

<u>Diagnosis</u>

- What was the experience of getting a diagnosis like, for you?
  - How did you receive confirmation of diagnosis (which professional or service)?
  - What was the GPs/Neurologists initial response – (what were you told? how did you feel about that…?)
- Do you feel you got sufficient information about your dementia at diagnosis?
- What was the most difficult aspect for you of obtaining a diagnosis?
  - any services that worked well/did not work so well? Inaccessible services/age restrictions?
- What could have made this period of time around diagnosis easier for you?

<u>Final questions</u>

- Is there anything else you would like add? Something we did not mention?
- **For the pilot**: This was the first in a number of interviews. Is there anything you think we could improve or change?

Semi-structured interview schedule for <u>caregiver</u> of person living with YOD

<u>Before diagnosis</u>

- Please can you tell me about the time leading up to diagnosis?
  - When did you first notice your relative was showing signs and symptoms of dementia?
  - What were the signs/symptoms that made you concerned/aware initially?
  - What did you do first (who did you tell, where did you go, etc?)

<u>Diagnosis</u>

- Can you tell me about the experience of getting the diagnosis confirmed?
  - How was diagnosis confirmed (responses from professionals or services)?
  - What initial services were utilised? (i.e. GP, consultant)

- What was the length of time between first symptoms and diagnosis (How simple was this process? how did you feel about this?)

- Were you satisfied with the amount of information your family received at the time of diagnosis?
    - What were you told? What was your relative told?
    - Any information you needed at that time, which was not available? Specify?
- Any gaps in services relating to diagnosis that did not work so well? (prompts; inaccessible services/age restrictions?)
- How did the news of the diagnosis impact on you?
    - How did the news of the diagnosis impact on other family members? Impact on immediate and wider family/life?
- Looking back to the time of your relative's diagnosis, is there anything that could have been done that would have made the experience better for you and your relative? (E.g. timing, information, advice etc)

Final questions

- Is there anything else you would like to tell us? Something we did not speak about?
- **For the pilot**: This was the first in a number of interviews. Is there anything you think we could improve or change?

Semi-structured interview schedule for <u>Health and Social Care Professionals</u>

- What type of diagnostic services do you provide to people suspected of having younger onset dementia?
- How many people with suspected young onset dementia are typically seen by you each month?
- How many people do you diagnose with younger onset dementia on a monthly basis?
- Who refers people with suspected younger onset dementia to your services?
- Who are the key personnel involved in the diagnosis process?
    - e.g. Geriatrician, Neurologist, GP, Old Age Psychiatrist, Neuro-Psychologist and other?
- What information do you typically give around diagnosis?
- What for you are the real barriers in attempting to diagnose dementia in people who belong to this age cohort?
- What might help you more easily diagnose dementia in people belonging to this age cohort
    - Easier access to other specialists
    - Easier access to diagnostic equipment
    - Better training in dementia
    - Age criteria review
    - Other (specify)
